# Anthracycline-Induced Cardiomyopathy: Prevalence and Risk Factors Among Pediatric Cancer Patients in a Tertiary Care Center in Jeddah, Saudi Arabia

**DOI:** 10.7759/cureus.81704

**Published:** 2025-04-04

**Authors:** Nouf Alghaith, Lena Afif, Ragheed A Justanieah, Sereen Alharbi, Rahaf Waggass, Daniah Abdullatif, Mohammed E Ahmed, Syed Faisal Zaidi

**Affiliations:** 1 Department of Pediatric Cardiology, King Saud Bin Abdulaziz University for Health Sciences, Jeddah, SAU; 2 Department of Pediatric Hematology Oncology, King Abdulaziz Medical City Ministry of National Guard Health Affairs, Jeddah, SAU; 3 Biostatistics, King Abdullah International Medical Research Center, Jeddah, SAU; 4 Integrative/Complementary Medicine, King Saud Bin Abdulaziz University for Health Sciences, Jeddah, SAU; 5 Integrative/Complementary Medicine, Faculty of Eastern Medicine, Hamdard University, Islamabad, PAK

**Keywords:** anthracycline, cardiomyopathy, cardiotoxicity, chemotherapy, pediatrics

## Abstract

Chemotherapy-induced cardiomyopathy (CCMP) is one of the well-defined toxicities associated with chemotherapy use that can lead to serious side effects. An example of a chemotherapeutic drug class that has been well documented over the years to cause CCMP is anthracyclines. To date, few studies have been carried out in Saudi Arabia on the prevalence of CCMP and the associated risk factors. Therefore, the objective of our research is to measure the prevalence and determine the risk factors of such phenomena. This is a comparative cross-sectional study. Data from 114 patients was retrieved from the medical records of the cardiac department at Princess Noorah Oncology Center, King Abdulaziz Medical City. The research included pediatric oncology patients aged 14 or under who were treated with anthracyclines from June 2016 to May 2024. We excluded patients who did not undergo ECHO. A consecutive sampling technique was used to collect the patients. Over the eight-year study period, we found that 7.34% (8/109) of the cohort developed CCMP, with a mean age at diagnosis of 6.39 ± 3.81 years. The mean dose of anthracycline received until the diagnosis of CCMP was 194.77±145.92 mg, with a median interval between anthracycline initiation and CCMP diagnosis of 13.65 (3-89) weeks. A significant association was found between thromboembolism, PDA, type of cancer, and the development of CCMP. We found that traditional predictors such as gender, age at diagnosis, and cumulative anthracycline dose were not predictors of CCMP.

## Introduction

Chemotherapy is a type of standard cancer therapy that targets different phases of the cell cycle; thus, it damages rapidly growing cells [1]. However, one of the major complications of anticancer treatment is that as it destroys cancerous cells, it also affects normal cells, which leads to the potential to produce toxicity [1]. A previous study proved that such toxicities could affect vital organs such as the kidneys, liver, lungs, and heart [2]. One of the well-defined toxicities strongly associated with chemotherapy is chemotherapy-induced cardiomyopathy (CCMP). CCMP is a range of conditions that can vary from a subtle decrease in systolic function, as seen in the left ventricular ejection fraction (LVEF), to the progression into heart failure (HF) with noticeable symptoms and clinical signs [3]. CCMP is a serious side effect that might limit the clinical use of chemotherapeutic agents, and eventually leads to an even worse prognosis than that of the underlying malignancy [4,5].

The degree of cardiotoxicity associated with chemotherapy is based on the cancer treatment, duration of therapy, and the risk factors presented in the patient [6-8]. An example of a chemotherapeutic drug class that has been well documented over the years to cause CCMP is anthracyclines, with incidence ranging between 3% and 48% depending on the cumulative dose received [7]. Furthermore, large studies have found that cumulative doxorubicin (an anthracycline) at a dosage of 250 mg/m² results in CCMP in 10% of patients; at 300 mg/m², 16% develop CCMP; at 400 mg/m², 32%; and at 550 mg/m², 65% [9]. CCMP or left ventricular dysfunction is defined as a drop in shortening fraction (FS%) below 29%. Therefore, anthracyclines can cause severe left ventricular dysfunction even at the lowest dose [10].

In addition to the cumulative dose being an essential determinant of the risk of cardiomyopathy, age is another modifying factor [7]. Patients older than 65 years as well as children have a higher risk of cardiotoxicity than young or middle-aged patients [7, 11]. Ganatra et al. mentioned that up to one-third of childhood cancer survivors end up developing various forms of cardiomyopathy, including heart failure, compared to 9% of adult breast cancer patients, despite both populations being on regimens including anthracyclines [12]. 

Moreover, studies on sex as a risk factor for CCMP have yielded conflicting results. While gender’s role in developing cardiotoxicities is not yet fully understood, several clinical and preclinical trials have suggested that female sex may offer protection against various chemotherapy-induced cardiac dysfunctions [13, 14]. However, when younger populations were examined, the findings were reversed [15-17]. For example, a study by Lipshultz et al. involving 120 pediatric patients concluded that female sex was an independent risk factor for anthracycline-induced cardiac abnormalities [18]. Additionally, various other risk factors, including genetic susceptibility, have been linked to CCMP and warrant further investigation [2,5,6].

Even though the discovery of newer antineoplastic drugs has begun to limit the use of anthracyclines, particularly in patients with a high cardiovascular risk, anthracyclines remain a significant treatment in half of all breast cancer and two-thirds of all pediatric chemotherapy regimens [19]. As more childhood cancer survivors reach adulthood because of advances in oncology therapy, they become increasingly vulnerable to late and progressive anthracycline-induced cardiotoxicity [20]. Nonetheless, diagnostic criteria for early identification of cardiac dysfunction in children, adolescents, and young adults (ages 1-40 years) are not well established [20].

The lack of well-defined early detection criteria is due to limited research on detection and prevention. These types of research, globally and particularly in Saudi Arabia, are lacking, whereas most of the studies focused on multiple organ toxicities, not specifically the heart [2], or discussed the role of one chemotherapeutic agent [21]. Therefore, the aim of our research is to provide a better insight into the relationship between anthracycline and cardiomyopathy in the pediatric age group, which is an under-investigated population. Additionally, the research aims to measure the prevalence and determine the risk factors of such phenomena.

## Materials and methods

Study design and setting

The study was a comparative cross-sectional study using data from Princess Noorah Oncology Center, King Abdulaziz Medical City (cardiac department) in Jeddah, Saudi Arabia. This study design was chosen to determine the prevalence and characteristics of anthracycline-induced cardiomyopathy in children with oncology diseases undergoing chemotherapy using patient medical records in the BestCare system and Xcelera program.

Study subjects

Our inclusion criteria included both groups (CCMP and non-CCMP), the patients who were treated with anthracyclines, and patients who were ≤14 years old. The CCMP group included patients who underwent an ECHO and were admitted to the hospital from June 2016 to May 2024. Our only exclusion criteria were developing cardiomyopathy post-cancer diagnosis, but before commencing anthracycline treatment. This was introduced after seeing such cases during data collection.

Sampling technique and study instrument

A consecutive sampling technique was applied to select the patients. Of all pediatric oncology patients from June 2016 to May 2024, 114 of them were treated with anthracyclines; however, five of them were excluded due to their ineligibility according to our inclusion criteria. The electronic best-care chart abstractions were reviewed by the pediatric cardiology consultant and pediatric hematology-oncology assistant consultant for accuracy.

The patients were divided into two groups: the patients who developed CCMP and the patients who did not develop CCMP. The collected data from both groups included numerical data: (patients’ age, total dose of anthracycline received, and shortening fraction “FS%”), and categorical data: (patients’ gender, diagnosis, e.g., CCMP or No CCMP, cancer type, stage, and chemotherapy protocol). Furthermore, additional data were collected for patients who developed cardiomyopathy, which are: (time interval between starting chemotherapy and development of cardiomyopathy, and total dose of Anthracycline at time of development of cardiomyopathy).

Data analysis

Parametric (mean and standard deviation) and non-parametric (median and interquartile range) approaches were used to describe the numerical data (age at diagnosis, total dose of anthracycline received, treatment duration, FS%, time interval between starting chemo and development of cardiomyopathy, and total dose of anthracycline at time of development of cardiomyopathy). Percentages and frequencies were used to describe categorical variables (gender, type of cancer, cancer stage, metastasis, chemotherapy regimen, diagnosis of CCMP, and comorbidities). Fisher's exact test was used to compare categorical data. Meanwhile, Wilcoxon one-way (Mann-Whitney) was used to compare categorical with continuous non-parametric data. A p-value less than 0.05 was considered statistically significant. The data was analyzed using the JMP PRO software, version 17.0 (SAS Institute Inc., Cary, NC).

Ethical considerations

No consent form was needed. This study used a chart review for data collection. Participants’ privacy and confidentiality were assured by giving each patient who met the inclusion criteria a serial numerical identifier from 1-109, and we referred to that number for the rest of the research to ensure anonymity. The original list that states each patient’s serial number and all data, hard and soft copies, was kept in a secure place within National Guard Hospital (NGHA) premises and accessed by the research team only. This study was approved by the King Abdullah International Medical Research Center (KAIMRC) with the IRB approval number SP21J/132/03.

## Results

Between June 2016 and June 2024, a total of 114 pediatric patients were diagnosed with cancer and received anthracyclines as part of their treatment protocol at Princess Noorah Oncology Center, Jeddah. Of the 114 patients, five did not meet the criteria of at least having one echo done; therefore, they were excluded from the study. On the other hand, two patients developed cardiomyopathy before the initiation of treatment with anthracyclines; thus, they were included as non-CCMP patients. The remaining 109 eligible patients were further divided into CCMP and non-CCMP groups.

The mean age of diagnosis for the total population was 6.39 years (standard deviation, SD 3.81). More than half of the cohort (57.80%) were males and 42.20% were females. Table 1 presents the demographic and clinical characteristics of the study population in general, stratified by the development of CCMP. Sixteen types of cancer were identified among the patients, with pre-B cells acute lymphoblastic leukemia (Pre-B ALL) preponderance (n=46, 42.20 %), followed by T-cell acute lymphoblastic leukemia (T-ALL), constituting 8.26 % (n=9). Furthermore, 21 (19.27 %) of the patients had metastatic cancer, 25 (22.94%) had localized cancer, and 63 (57.80 %) were not applicable due to the nature of the disease. Median total dose of anthracycline received by all the patients was 118.5 mg (interquartile range, IQR 0.46-733.08 mg) in a treatment duration of 38 weeks (IQR 1.43-216 weeks). A total of 8 (7.34%) out of 109 patients were diagnosed with CCMP; their characteristics are mentioned in Table 2. The CCMP group consisted of two males (25 %) and six females (75 %), with the mean age at diagnosis of 7.00 years (SD ±3.16 years). Wilms tumor was the most prevalent cancer among the CCMP group (n=3, 37.5%), and metastasis was found to be present in half of this group (n=4, 50%).

**Table 1:** Demographics and characteristics of the study cohort. N/A represents certain cancer types that either do not have a staging system or cannot be metastasized. SR> HR represent transformation of standard-risk ALL (WBCs at the time of the diagnosis is less than 50,000 cell/mm3) to high-risk ALL (WBCs at the time of the diagnosis is more than 50,000 cell/mm3). HR> SR represent transformation of high-risk ALL to standard-risk with isolated symptomatic early CNS relapse. Group B and C represent risk stratification by Berlin Frankfurt Munster (BFM) in Burkitt lymphoma. n: number (sample size); CCMP: chemotherapy-induced cardiomyopathy; SD: standard deviation; IQR: interquartile range; pre-B ALL: Pre-B cell acute lymphoblastic leukemia; T-ALL: T-LBLT cell acute lymphoblastic leukemia; T-LBL: T cell lymphoblastic lymphoma; AML: acute myeloid leukemia; APL: acute promyelocytic leukemia; SR: standard-risk; HR: high-risk; CNS: central nervous system

Characteristics	Overall (n=109)	Non-CCMP group (n=101)	CCMP group (n=8)
Prevalence, n (%)	109	101 (92.66%)	8 (7.34%)
Demographics			
Gender, n (%)			
Male	63 (57.80)	61(60.40)	2 (25)
Female	46 (42.20)	40 (39.60)	6 (75)
Clinical features			
Age at diagnosis, years (mean± SD)	6.39±3.81	6.34±3.86	7±3.16
Total anthracycline dose, mg (median (IQR)	118.5 (0.46-733.08)	112 (0.46-733.08)	209.27 (68.85-487.32)
Treatment duration, weeks (median (IQR)	38 (1.43-216)	38 (1.43-216)	35.5 (24.5-61)
Comorbidities, n (%)			
Yes	44 (40.37)	40 (60.39)	6 (75)
No	65 (59.63)	61 (39.60)	2 (25)
Cancer type, n (%)			
pre-B ALL	46 (42.20)	45 (44.55)	1 (12.5)
T-ALL	9 (8.26)	9 (8.91)	
T-LBL	2 (1.84)	2 (1.98)	
Hodgkin’s lymphoma	5 (4.59)	5 (4.95)	
Wilms tumor	8 (7.34)	5 (4.95)	3 (37.5)
Rhabdomyosarcoma	4 (3.76)	4 (3.96)	
Early metastatic recurrent Ewing sarcoma	1 (0.92)	1 (0.99)	
Ewing sarcoma	7 (6.42)	6 (5.94)	1 (12.5)
Pancreatic myeloid sarcoma	1 (0.92)	1 (0.99)	
Undifferentiated sarcoma	3 (2.75)	2 (1.98)	1 (12.5)
Neuroblastoma	7 (6.42)	7 (6.93)	
Osteosarcoma	2 (1.84)	1 (0.99)	1 (12.5)
AML	3 (2.75)	2 (1.98)	1 (12.5)
Burkitt lymphoma	8 (7.34)	8 (7.92)	
Infantile ALL	2 (1.84)	2 (1.98)	
APL	1 (0.92)	1 (0.99)	
Cancer stage, n (%)			
I	1 (0.92)	1 (0.99)	
II	2 (1.83)	1 (0.99)	1 (12.5)
III	3 (2.75)	3 (2.97)	
IV	7 (6.42)	3 (2.97)	4 (50)
IA	3 (2.75)	3 (2.97)	
IR	2 (1.83)	2 (1.98)	
2A	2 (1.83)	2 (1.98)	
SR	26 (23.85)	25 (24.75)	1 (12.5)
HR	32 (29.35)	31 (30.69)	1 (12.5)
SR > HR	9 (8.26)	9 (8.91)	
Group C	6 (5.50)	6 (5.94)	
HR > isolated symptomatic early CNS relapse (HR > SR)	1 (0.92)	1 (0.99)	
Group B	1 (0.92)	1 (0.99)	
N/A	14 (12.84)	13 (12.87)	1 (12.5)
Metastasis, n (%)			
Yes	21 (19.27)	17 (16.83)	4 (50)
No	25 (22.94)	23 (22.77)	2 (25)
N/A	63 (57.80)	61 (60.40)	2 (25)

**Table 2:** Characteristics of the eight study participants with CCMP (CCMP group). FS% (fractional shortening) measures left ventricular function.

Characteristics	No. of patients
Total dose of anthracycline received until CCMP diagnosis, mg	194.77±145.92
Interval between anthracycline initiation and CCMP diagnosis, week(s)	13.65 (3-89)
Echocardiographic parameters, (mean± SD)	
FS%, baseline (%)	35.08±5.54
FS%, mid-treatment/at diagnosis (%)	24.71±6.91
FS%, last (%)	29.21±6.27

**Figure 1:**
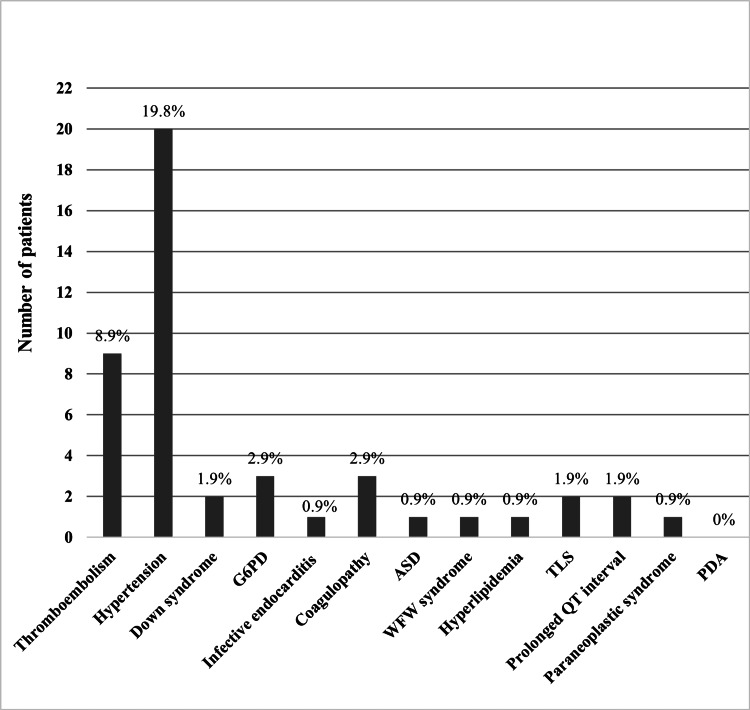
Comorbidities of non-CCMP group interpreted in frequencies and percentages. ASD: atrial septal defect; WPW: Wolff-Parkinson-White; TLS: tumor lysis syndrome; PDA: patent ductus arteriosus

**Figure 2:**
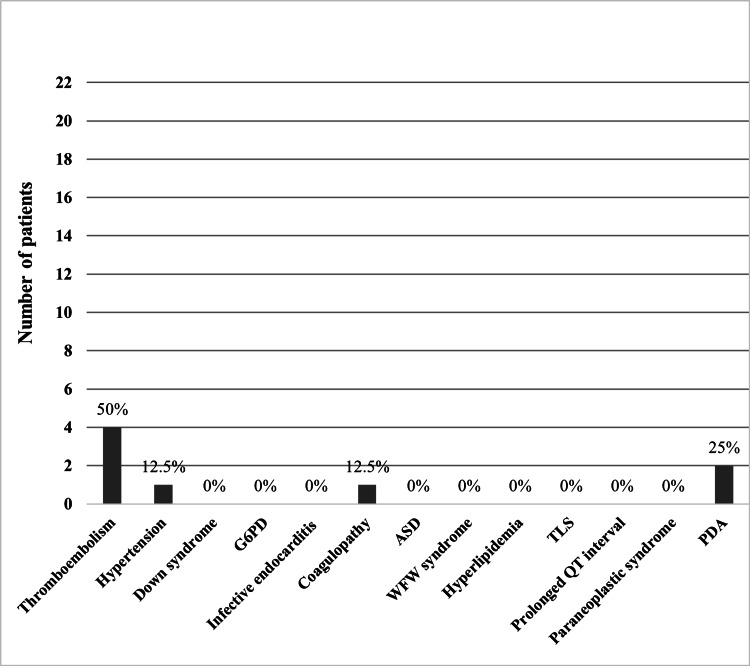
Comorbidities of CCMP group interpreted in frequencies and percentages. ASD: atrial septal defect; WPW: Wolff-Parkinson-White; TLS: tumor lysis syndrome; PDA: patent ductus arteriosus

A total of 44 (44/109, 40.37%) participants had comorbidities, while 65 (65/109, 59.63%) did not. As seen in Figure 1, the most common comorbidity in the non-CCMP group was hypertension (19.8%), followed by thromboembolism (8.9%) as the second most common. As of the CCMP group, the most prevalent comorbidity was thromboembolism (50%), followed by PDA (25%), in Figure 2. The rest of the variables are in Table 3. 

**Table 3:** Analysis of the CCMP group by study variables. Data are reported as mean ± SD and n (%), unless otherwise stated. Fisher's exact test was used for categorical data, and the Wilcoxon rank-sum test (Mann-Whitney U test) was used for comparisons between categorical and continuous non-parametric data. *Statistically significant at less than 5% ASD: atrial septal defect; PDA: patent ductus arteriosus; WPW: Wolff-Parkinson-White; IE: infective endocarditis

Variables	Non-CCMP group (n=101)	CCMP group (n=8)	p-value
Gender			
Male	61 (96.83)	2 (3.17)	0.068
Female	40 (86.96)	6 (13.04)	0.068
Age at diagnosis, years (mean±SD)	6.34±3.86	7±3.16	0.64
Total anthracycline dose, mg	152.29±130.38	223.94±132.06	0.14
Treatment duration, weeks	44.30±33.84	38.31±11.89	0.62
Thromboembolism	9 (69.23)	4 (30.77)	0.007*
Hypertension	20 (95.24)	1 (4.76)	1.00
PDA	0 (0.0)	2 (100.0)	0.005*
ASD	1 (100.0)	0 (0.0)	1.00
Prolonged QT interval	2 (100.0)	0 (0.0)	1.00
WPW syndrome	1 (100.0)	0 (0.0)	1.00
IE	1 (100.0)	0 (0.0)	1.00

## Discussion

Anthracyclines are one of the commonly used and well-defined chemotherapeutic agents that can cause CCMP, especially in pediatrics [7]. The incidence of cardiomyopathy induced by anthracyclines ranges from 3% to 48% depending on the total dose of the drug [7]. This study aimed to focus on the relationship between anthracycline and cardiomyopathy in pediatric oncology patients to measure the prevalence and risk factors of CCMP among the population, in addition to their outcomes post-diagnosis.

The study demonstrated a correlation between CCMP and multiple demographics, including age and gender. We found that 7.34% of our pediatric patients developed CCMP, a figure consistent with the reported range of 5-10% in previous studies [16, 19], which correlates with one well-known risk factor for CCMP: extremes of age

Extremes of age are usually defined as either <18 years or ≥80 years at the time of treatment [20, 21]. One study mentioned that pediatric patients who received anthracyclines are 15 times more likely to develop heart failure than the general population and eight times more likely to die from cardiovascular diseases [21]. The mean age at diagnosis for the CCMP group in our study was slightly higher (7.00 years) than the overall cohort (6.39 years), suggesting a possible trend towards older age at diagnosis being linked to a higher CCMP risk, although age was not a statistically significant factor in our analysis.

On the other hand, the literature was not as consistent in regards of gender, as it presents conflicting information on how gender relates to CCMP development. Jain et al. [22] noted that male sex significantly increases the risk of heart failure in various cardiovascular conditions, including dilated cardiomyopathy. Conversely, Loar et al. [23] suggested that female sex might contribute to cardiotoxicity. Some studies, like Mulrooney et al. [24], have found no significant gender differences in CCMP incidence. In our study, CCMP patients were mostly female (75%), but gender did not emerge as a significant risk factor in our cohort. This underscores the need for further investigation in larger, more diverse populations.

Several potential risk factors were investigated in the study, such as the cancer type, treatment protocol, medical history of certain comorbidities, and related cardiological conditions.

The predominance of Wilms tumor in the CCMP group (37.5%) aligns with literature indicating that certain cancer types may have a higher propensity for treatment-related cardiotoxicity [25].

Regarding chemotherapy regimens, the most frequent regimen used was CALL 17 HR (in 22 out of 109 patients). Among CCMP patients, specific regimens included COG ARST0332 (one patient), PET09 (two patients), one of whom received it as a second regimen with COG ARST0332, CALL 17 IR with a second regimen AML 09 (one patient), CALL 17 HR (one patient), WT09 AVD regimen 5 (in three patients), and EPCD regimen 6 (in two patients) as a second chemo regimen in patients who had received WT09 AVD regimen 5. Most of these regimens contain doxorubicin, which is highly associated with the development of CCMP [20, 21].

The mean cumulative dose of anthracyclines received by CCMP patients was higher (194.77 mg) compared to the overall cohort; however, the cumulative dose did not emerge as a statistically significant risk factor. This finding diverges from several studies that have established a dose-dependent relationship with CCMP [26]. The variability in anthracycline dosing protocols and patient-specific factors may account for this discrepancy. Our population diverges from prior cohorts in two key aspects. First, regional protocols adapted for the local population like WT09 AVD (used in 37.5% of CCMP cases) employ shorter, higher-intensity anthracycline cycles, potentially altering toxicity profiles [27]. Second, genetic homogeneity due to consanguinity (~50% in Saudi Arabia) [28] may elevate susceptibility to congenital anomalies (e.g., PDA) and chemotherapy-induced cardiotoxicity.

Our findings contrast with established literature identifying cumulative anthracycline dose, age, and gender as predictors of CCMP [6, 26]. The lack of statistical significance for these variables in our cohort may reflect limited power due to the small sample size (n=8 CCMP cases), which reduces sensitivity to detect modest associations. For instance, the median cumulative anthracycline dose in our population (118.5 mg) was substantially lower than thresholds associated with cardiotoxicity in adult studies (≥250 mg/m²) [8, 26]. Additionally, regional treatment protocols, such as shorter cycles in regimens like CALL 17 HR, may mitigate cumulative toxicity. Unmeasured confounders, including genetic polymorphisms (e.g., RARG or CBR3) [29] or concurrent cardioprotective agents (e.g., dexrazoxane), could further obscure dose-dependent relationships.

As DVT and hypertension were the most common co-morbidities among our patients, a correlation analysis with the incidence of CCMP was performed. Significant risk factors for CCMP development identified in our study included thromboembolism, with a notable 50% of CCMP patients having thromboembolism compared to non-CCMP patients. This finding is particularly relevant as it highlights the need for vigilant monitoring and management of thromboembolic events in pediatric cancer patients receiving Anthracyclines. Previous studies also corroborated the notion of the cardiotoxic potential related to thromboembolic events in this population [30].

Thromboembolism emerged as a critical risk factor for CCMP (30.77% vs. 8.91%, p=0.007), likely due to microvascular injury from hypercoagulable states amplifying anthracycline toxicity [30]. To translate this into practice, we propose: (1) prophylactic anticoagulation for high-risk patients (e.g., metastatic disease or inherited thrombophilia), (2) serial echocardiography paired with biomarkers (troponin, BNP) during therapy, and (3) risk stratification models integrating thromboembolism history, PDA, and cancer type (e.g., Wilms tumor) to prioritize surveillance [31].

Moreover, there is one study that considered hypertension a complication induced by anthracycline [32], whereas another study considered it as a risk factor [23]. However, we did not find any significant association between CCMP and hypertension among our study sample, and most of them ended up with resolved hypertension.

Furthermore, we investigated the correlation between CCMP and various cardiological findings that could easily be screened during the treatment period in the effort to develop better guidelines for early detection of CCMP. The findings are prolonged QT interval, patent ductus arteriosus (PDA), Wolff-Parkinson-White (WPW) syndrome, and infective endocarditis. Only patent ductus arteriosus (PDA) had a significant association. The inclusion of PDA in our analysis was informed by clinical observations during echocardiographic screenings, where incidental PDA findings were noted in patients who later developed CCMP. To investigate potential associations, we conducted a Fisher’s exact test, which revealed a statistically significant link between PDA and CCMP (p=0.005), with 100% of PDA cases (2/2) occurring in the CCMP group. While no prior studies have reported this association [17, 33], we hypothesize that hemodynamic stress from persistent left-to-right shunting in PDA may exacerbate anthracycline-induced myocardial injury. Specifically, chronic volume overload in the left ventricle could amplify oxidative stress, a key mechanism of anthracycline cardiotoxicity [34, 35]. Additionally, shared genetic susceptibilities, such as polymorphisms in redox-regulating genes (e.g., NQO1) or angiogenesis pathways (e.g., VEGF), might predispose patients to both congenital heart defects and chemotherapy-related cardiac dysfunction [29, 34].

Interestingly, prolonged QT interval, Wolff-Parkinson-White syndrome, and infective endocarditis were not significantly associated with CCMP in our study. ECG changes, such as tachycardias, can occur in 20% to 30% of CCMP patients. A prolonged QT interval is considered a transient electrophysiological (ECG) abnormality after starting treatment and may indicate pericarditis, myocarditis syndrome, or acute or subacute left ventricular failure [33-36]. Other than QT prolongation, CCMP patients might also show nonspecific ST- and T-wave changes, T-wave flattening, and decreased QRS voltage on the ECG [36, 37]. These findings can aid in the early detection of CCMP, although they are not the gold standard for diagnosing the disease.

WPW syndrome is a condition characterized by an abnormal electrical pathway in the heart that can lead to episodes of tachycardia. While WPW can complicate cardiac function, its lack of significant association with CCMP in our cohort suggests that the presence of this syndrome does not inherently increase the risk of anthracycline-induced cardiotoxicity. This finding contrasts with some hypotheses that pre-existing electrical abnormalities might predispose patients to higher cardiotoxic risk [38].

Similarly, infective endocarditis, an infection of the heart valves or endocardium, was not significantly associated with the development of CCMP. This lack of association might indicate that while infective endocarditis is a severe condition requiring intensive management, it does not necessarily exacerbate the cardiotoxic effects of Anthracyclines. This is consistent with other studies that have not found a direct link between endocarditis and chemotherapy-induced cardiotoxicity, suggesting that the mechanisms of cardiotoxicity may be more related to direct myocardial damage from Anthracyclines rather than secondary infections [39].

As for our patients’ outcomes, one of our CCMP patients unfortunately passed away, though not because of cardiomyopathy. She was a nine-year-old girl who had been admitted to the hospital for six months due to severe fatigue and pallor. Three weeks before she passed, her fatigue and pallor worsened, and she began experiencing bone pain. She was later diagnosed with pancytopenia and a dental abscess. She was then transferred to the ICU with a low-grade fever and received packed RBCs, tazobactam, vancomycin, and intravenous allopurinol. Despite all interventions, she went into septic shock and died. Additionally, she was on vasopressin for her cardiomyopathy.

Another CCMP patient, on the other hand, showed improvement. She has been on anti-failure medications for four years, including enoxaparin, digoxin, furosemide, captopril, cholecalciferol, and low molecular weight heparin due to a previous history of thromboembolism in the medial cerebral artery. However, over the last two months, her condition deteriorated due to non-compliance with her medications, causing her (FS) to decrease from 26% to 22-25%. Despite this, her overall condition remains stable, with no serious complications.

For the remaining three CCMP patients, they all had transient CCMP, which was defined as low FS% without overt heart failure. All three patients recovered fully without any lasting cardiac dysfunction.

Limitations

The retrospective nature of this study introduces potential biases. Selection bias may arise from excluding patients without echocardiographic follow-up (n=5), potentially underrepresenting asymptomatic or mildly symptomatic CCMP cases. For instance, patients lost to follow-up due to treatment discontinuation or death might have had undiagnosed cardiomyopathy, skewing prevalence estimates downward. Information bias could result from incomplete documentation of comorbidities (e.g., transient hypertension or subclinical thromboembolism) or variations in echocardiographic reporting practices over the eight-year study period. To mitigate this, data abstraction was performed by pediatric cardiology and oncology consultants using standardized criteria, and ambiguous records were excluded (n=2 with pre-existing cardiomyopathy).

The limited number of CCMP cases (n=8) reduces statistical power to detect modest associations (e.g., age, cumulative dose) and increases the risk of Type II errors. Subgroup analyses, such as gender-specific risk (two males vs. six females), are unreliable due to insufficient sample diversity. To address this, non-parametric tests (e.g., Mann-Whitney U) were prioritized for skewed data, and results were interpreted conservatively, emphasizing effect sizes over p-values.

Key confounders such as socioeconomic status, nutritional status, and concurrent cardioprotective therapies (e.g., dexrazoxane) were not analyzed. For example, malnutrition, prevalent in pediatric oncology populations, may exacerbate anthracycline toxicity by impairing antioxidant defenses [35]. Similarly, genetic polymorphisms (e.g., RARG or CBR3) [29] or concurrent medications (e.g., trastuzumab) could modulate cardiotoxicity risk but were not assessed.

Findings are most applicable to pediatric populations receiving similar anthracycline protocols (e.g., CALL 17 HR) in tertiary care settings. Regional factors, such as high consanguinity rates (~50% in Saudi Arabia) [28] and unique genetic predispositions, may limit generalizability to ethnically diverse cohorts. Additionally, lifestyle factors (e.g., physical activity, environmental exposures) and long-term medication adherence were not evaluated, though these could influence late-onset cardiotoxicity.

## Conclusions

This study contributes to the existing body of knowledge by identifying specific risk factors associated with CCMP in pediatric cancer patients. The significant association with cancer type, thromboembolism, and PDA highlights the need for proactive cardiac monitoring and intervention strategies. Our findings underscore the importance of individualized treatment plans and vigilant follow-up to mitigate the risk of cardiotoxicity. For these populations, we recommend baseline echocardiography prior to anthracycline initiation, followed by serial assessments every three months during therapy and annually thereafter. Declines in shortening fraction (FS%) ≥10% from baseline should prompt intensified monitoring,

Future studies should prioritize multicenter cohorts to validate PDA and thromboembolism as risk factors, mechanistic investigations of genetic or inflammatory pathways (e.g., RARG or VEGF signaling), and longitudinal designs to assess late-onset toxicity. Our findings are most generalizable to pediatric populations receiving similar regimens (e.g., CALL 17 HR); extrapolation to adults or regions with divergent protocols requires caution.
